# Dietary Index for Gut Microbiota and Risk of All-Cause and Cardiovascular Mortality Across Cardiovascular-Kidney-Metabolic Syndrome Stages 0–3: A Nationwide Prospective Cohort Study

**DOI:** 10.31083/RCM45493

**Published:** 2026-02-13

**Authors:** Yifei Wang, Aodi Huang, Lei Bi, Siyuan Li, Qing Li, Ping Zhang, Tingting Lv

**Affiliations:** ^1^Department of Cardiology, Beijing Tsinghua Changgung Hospital, Tsinghua Medicine, Tsinghua University, 102218 Beijing, China; ^2^School of Clinical Medicine, Tsinghua University, 100084 Beijing, China

**Keywords:** dietary index for gut microbiota, cardiovascular-kidney-metabolic syndrome, mortality, inflammation, NHANES

## Abstract

**Background:**

Cardiovascular-kidney-metabolic (CKM) syndrome represents a progressive disorder characterized by the interplay of cardiovascular pathologies, chronic renal impairment, and metabolic dysregulation. Therefore, this study aimed to examine the relationship between the dietary index for gut microbiota (DI-GM) and mortality outcomes, including both all-cause and cardiovascular-specific mortality, in individuals classified with CKM syndrome stages 0–3.

**Methods:**

Our study cohort consisted of 7884 adult participants aged 30–79 years from the National Health and Nutrition Examination Survey (NHANES) conducted between 2007 and 2018. Dietary intake data obtained through 24-hour dietary recalls and food frequency questionnaires were used to calculate the DI-GM scores, incorporating both components beneficial to the microbiota and those with potentially detrimental nutritional effects. The primary and secondary endpoints were all-cause mortality and cardiovascular-related mortality, respectively. The Kaplan–Meier survival analysis, Cox proportional hazards regression models, and restricted cubic spline (RCS) techniques were employed in the statistical analyses.

**Results:**

The participants had a median age of 50 years, with females comprising 52.97% of the cohort. Over a median follow-up period of 77 months, we documented 469 all-cause deaths (4.56%) and 105 cardiovascular fatalities (1.02%). Elevated beneficial scores for the DI-GM demonstrated significant inverse associations with both all-cause (*p* < 0.001) and cardiovascular mortality (*p* = 0.017). However, while the total DI-GM scores showed correlation with decreased all-cause mortality (*p* < 0.001), no significant association emerged for cardiovascular mortality. Following the employment of a comprehensive adjustment, the hazard ratio (HR) for the total DI-GM score and all-cause mortality was 0.90 (95% confidence interval (CI): 0.82–0.98). For the beneficial components, the HR was 0.88 (95% CI: 0.79–0.98) for all-cause mortality and 0.87 (95% CI: 0.77–0.99) for cardiovascular mortality. RCS modeling revealed a U-shaped correlation between the total DI-GM scores and all-cause mortality, which was in contrast to a linear association for the beneficial scores. The systemic inflammation index (SII) accounted for 5.29% and 8.45% of the observed associations between the total and beneficial DI-GM scores and all-cause mortality, respectively.

**Conclusions:**

Elevated DI-GM dietary scores, particularly those emphasizing food components beneficial to the gut microbiota, demonstrate protective associations against both all-cause and cardiovascular mortality in individuals with CKM syndrome in stages 0–3. These protective effects appear partially influenced by systemic inflammatory pathways.

## 1. Introduction

In 2023, the American Heart Association (AHA) proposed a novel classification system termed cardiovascular-kidney-metabolic (CKM) syndrome [1]. This framework emphasizes the interrelated nature of cardiovascular disorders, chronic kidney impairment, and metabolic dysfunctions. The CKM model stratifies patients into five progressive phases (stage 0 through 4), with the latter two stages denoting advanced pathological conditions [2]. Epidemiological data from 2011–2020 revealed the following distribution among US adults: 10.6% in stage 0, 25.9% in stage 1, 49.0% in stage 2, 5.4% in stage 3, and 9.2% in stage 4 [3]. While stages 0–3 encompass subjects without clinically manifest cardiovascular disease, these categories demonstrate escalating hazards for future cardiovascular events and all-cause mortality [4, 5]. Early identification and targeted prevention are therefore critical for long-term health burden reduction.

Several factors influence CKM syndrome progression and mortality. Research indicates that certain demographic and socioeconomic factors, including being male, aged 65 or older, and of African American descent, correlate with an elevated likelihood of developing severe CKM syndrome [3]. In addition to these variables, health-related social determinants—specifically limited educational attainment and significant social risk exposure—have been shown to correlate with worse clinical outcomes [6, 7, 8]. Nevertheless, current scientific investigations have not sufficiently examined how different nutritional habits influence the advancement and clinical course of CKM syndrome.

Nutrition plays a crucial role in influencing metabolic and cardiovascular wellbeing. Different eating habits have been demonstrated to affect survival rates among patients diagnosed with disorders including diabetes, high blood pressure, and cardiovascular disease [9, 10, 11]. These effects are mediated through mechanisms like gut microbiota regulation, immune modulation, and antioxidant activity [12, 13, 14]. The dietary index for gut microbiota (DI-GM) was specifically designed to evaluate the impact of 14 distinct dietary elements on intestinal microbial health, classifying these components as either advantageous or detrimental [12]. Furthermore, this index has demonstrated significant associations with the development and progression of various chronic conditions, including cerebrovascular accidents, metabolic disorders, hepatic steatosis, and age-related health decline [15, 16, 17, 18]. Notably, while prior investigations have examined DI-GM in relation to diabetes and stroke, the current study focuses on individuals in CKM stages 0–3, a population characterized by the absence of overt cardiovascular disease but heightened susceptibility to long-term complications, thereby representing a crucial period for implementing preventive measures and assessing DI-GM’s predictive capacity.

Utilizing data from the National Health and Nutrition Examination Survey (NHANES) spanning 2007 to 2018, this investigation aims to examine the relationship between DI-GM (comprising aggregate, favorable, and adverse component scores) and mortality risk among subjects with CKM syndrome across stages 0 through 3. By identifying dietary patterns linked to CKM syndrome prognosis, the study seeks to inform potential nutritional strategies for preventing disease progression and reducing mortality in early-stage CKM syndrome populations.

## 2. Materials and Methods

### 2.1 Data Source and Study Population

The research employed data obtained from the NHANES spanning 2007 to 2018, which constitutes a nationally representative study administered collaboratively by the Centers for Disease Control and Prevention (CDC) and National Center for Health Statistics (NCHS). NHANES implements a complex sampling methodology involving multiple stages and stratification, gathering comprehensive health information from both adult and pediatric populations across the United States biennially. All investigative procedures received approval from the NCHS Research Ethics Review Board and strictly complied with Strengthening the Reporting of Observational Studies in Epidemiology (STROBE) reporting standards.

**Fig. 1.**
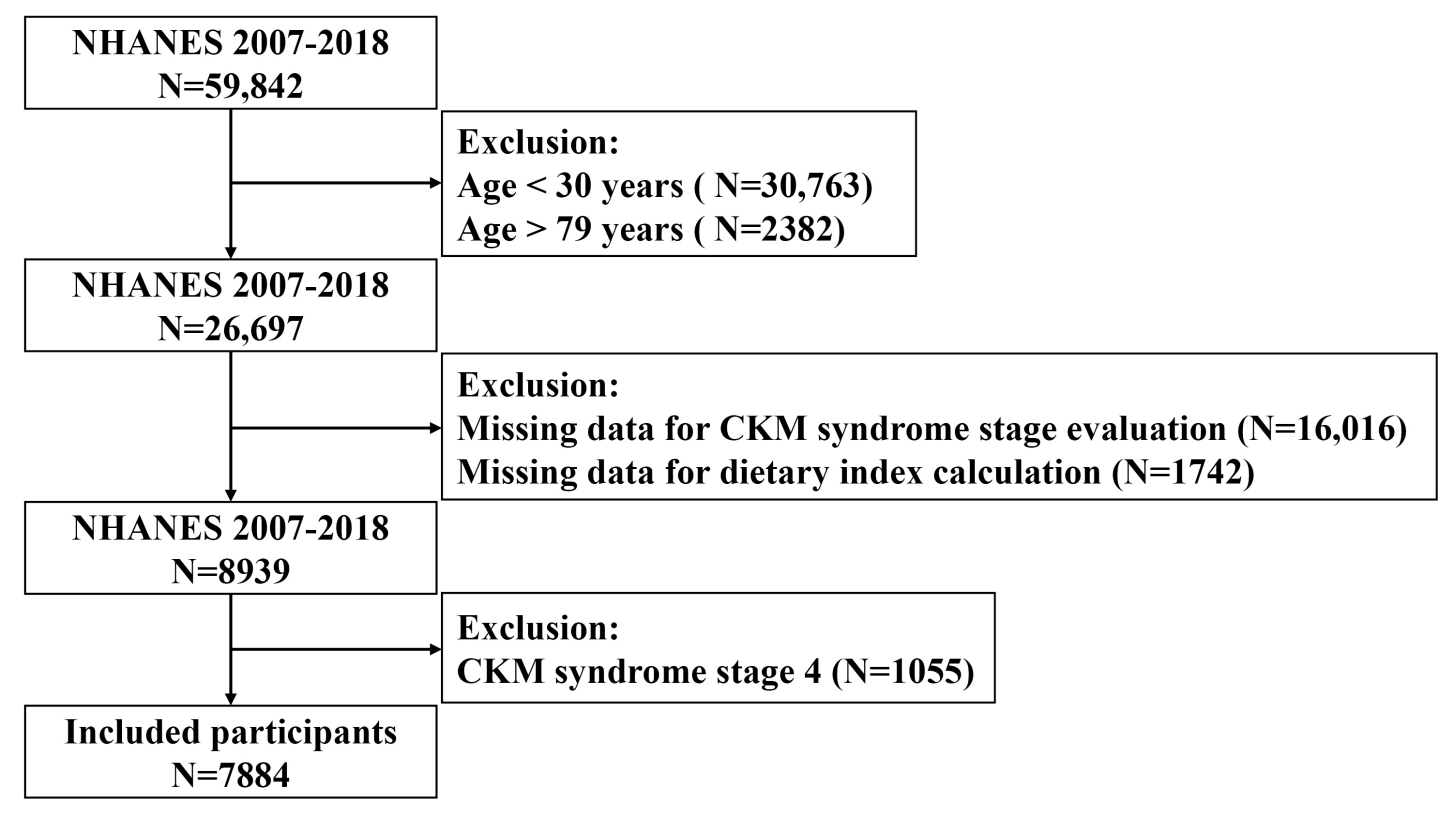
**Flowchart of study population inclusion and exclusion**. NHANES, National Health and Nutrition Examination Survey; CKM, cardiovascular-kidney-metabolic.

Participants aged 30 to 79 years were considered eligible for inclusion, consistent with the applicability of the PREVENT equations for cardiovascular risk estimation. Participants were excluded from the study if they did not have adequate data to determine their CKM syndrome stage, possessed incomplete dietary records necessary for computing DI-GM scores, or were diagnosed with CKM syndrome stage 4. Following these exclusion criteria, the final study cohort comprised 7884 individuals (Fig. 1). The NCHS Research Ethics Review Board granted approval for all NHANES study protocols, with each participant providing written consent prior to enrollment. This investigation adhered to the ethical principles outlined in the Declaration of Helsinki. Given that NHANES datasets are anonymized and accessible to the public, no further institutional review board authorization was deemed necessary.

### 2.2 CKM Syndrome Stage Evaluation

CKM syndrome stages (0–4) were defined based on criteria from Aggarwal *et al*. [3] and adapted for NHANES data using the classification system from Tang *et al*. [19] (detailed information listed in **Supplementary Table 1**) [20]. In brief, CKM syndrome stages were categorized as follows:

Stage 0: No health risk factors related to CKM syndrome.

Stage 1: Dysfunctional adiposity.

Stage 2: With metabolic risk factors (hypertension, diabetes, and dyslipidemia), or chronic kidney disease (CKD).

Stage 3: Subclinical CVD on top of stage 2 criteria (10-year CVD risk ≥20%).

Stage 4: Established CVD.

The assessment of CKM syndrome progression stages employed Predicting Risk of Events via Estimated Cardiac Trajectories (PREVENT) equations [21], a validated tool designed for estimating 10-year cardiovascular disease risk in the U.S. adult population aged 30–79 years. Chronic kidney disease staging was determined using the race-neutral creatinine-based equation developed by the Chronic Kidney Disease Epidemiology Collaboration (CKD-EPI, 2021).

### 2.3 DI-GM Calculation

The calculation of DI-GM scores utilized 24-hour dietary recall information obtained from NHANES, incorporating 14 distinct dietary elements according to the scoring methodology established by Kase *et al*. [12] (refer to **Supplementary Table 2**). Each study subject provided dietary data through two separate 24-hour recall sessions conducted on non-consecutive days: the initial assessment occurred during face-to-face interviews at the Mobile Examination Center, followed by a subsequent telephone-based recall conducted 3–10 days afterward. To minimize individual variability and obtain more representative dietary patterns, the present analysis employed averaged consumption values derived from both recall sessions for DI-GM computation. This dietary index comprises 14 components classified as either advantageous or detrimental to gut microbiota health. Positive dietary elements encompassed avocado, broccoli, chickpeas, coffee, cranberries, fermented dairy products, dietary fiber, soy products, and whole grains (though green tea consumption data were unavailable in NHANES). Negative components consisted of processed meats, red meats, refined grain products, and high-fat dietary patterns (defined as ≥40% of total energy intake).

Scoring methodology assigned 1 point when beneficial food consumption exceeded gender-specific median values or when unfavorable food intake fell below median levels. The comprehensive DI-GM scale spanned from 0 to 13 points, with beneficial components contributing 0–9 points and unfavorable components accounting for 0–4 points. Study participants were subsequently stratified into quartile groups according to their total scores and beneficial component scores.

### 2.4 Covariates and Exposure Variables

The selection of covariates was guided by existing research evidence, clinical importance, and statistical associations. These variables encompassed demographic characteristics (age, gender, racial background), socioeconomic indicators (educational attainment, marital situation, poverty-to-income ratio), lifestyle factors (tobacco use, alcohol intake), anthropometric measurements (body mass index), CKM syndrome severity, and biochemical parameters (serum uric acid levels and leukocyte counts). The poverty income ratio was calculated as household earnings relative to federal poverty guidelines and stratified into three categories: below poverty level (<1), moderate income (1–3), and higher income (≥5). Body mass index was derived from the formula of body weight in kilograms divided by the square of height in meters. The systemic inflammation index was determined through the equation: (platelet concentration × neutrophil count)/lymphocyte concentration [22]. Ethnic classification followed NHANES protocols, including Mexican American, other Hispanic populations, non-Hispanic Caucasian, non-Hispanic African American, and other racial groups. Educational background was dichotomized into incomplete secondary education versus secondary education completion or higher. Marital status was classified as either partnered (married/cohabiting) or unpartnered. Comorbid conditions were identified using established diagnostic criteria. Hypertension was diagnosed based on elevated blood pressure readings (systolic ≥140 mmHg or diastolic ≥90 mmHg), clinician-confirmed diagnosis, or antihypertensive medication use. Diabetes mellitus was defined by fasting glucose levels ≥126 mg/dL, glycated hemoglobin ≥6.5%, physician-documented diagnosis, or hypoglycemic drug therapy. Dyslipidemia criteria included total cholesterol ≥240 mg/dL, triglycerides ≥200 mg/dL, physician diagnosis, or lipid-modifying treatment. Chronic kidney disease was identified when the estimated glomerular filtration rate fell below 60 mL/min/1.73 m^2^ or the urinary albumin-to-creatinine ratio exceeded 30 mg/g, calculated using the race-neutral CKD-EPI (2021) formula.

### 2.5 Outcomes

The study’s principal endpoint encompassed mortality from any cause, while cardiovascular-related deaths constituted the secondary endpoint. Vital status and specific causes of death were determined by cross-referencing NHANES participants with publicly available mortality records from the National Death Index (NDI), with follow-up data extending until December 31, 2019. Deaths attributed to cardiovascular causes were classified according to International Classification of Diseases (ICD)-10 codes I00–I09, I11, I13, and I20–I51. Follow-up duration for each participant was calculated from their initial examination date until either their death or the study’s termination date, whichever occurred earlier.

### 2.6 Statistical Analysis

The statistical analyses incorporated NHANES’s sophisticated multistage probability sampling framework through the application of proper sampling weights, stratification variables, and primary sampling units, executed in R software (V4.4.2, R Foundation for Statistical Computing, Vienna, Austria) utilizing the survey package. In compliance with NHANES analytical protocols, descriptive statistics were presented as mean values with SE for normally distributed parameters, median values with interquartile range (IQR) for non-normally distributed variables, and frequency counts with weighted proportions for categorical measures. Comparative analyses between groups employed one-way ANOVA for normally distributed continuous data, Kruskal-Wallis tests for skewed continuous variables, and chi-square tests for categorical data comparisons.

The analysis of survival outcomes utilized Kaplan-Meier plots to evaluate variations in mortality across different DI-GM quartiles. Survey-adjusted Cox proportional hazards regression models were employed to accommodate NHANES’ intricate sampling methodology, generating hazard ratios (HRs) and corresponding 95% confidence intervals (CIs) for both all-cause and cardiovascular-related deaths. Covariates were chosen for model adjustment based on their statistical importance, clinical significance, and existing research evidence. To prevent excessive model adjustment, clinical parameters closely associated with CKM staging (including lipid profiles, blood pressure measurements, and glucose levels) were excluded, consistent with previous investigations [19, 20]. Due to significant data gaps in NHANES, factors such as exercise levels, caloric consumption, and pharmaceutical treatments (including antihypertensive, antidiabetic, and cholesterol-lowering medications) were omitted from the multivariate analyses. Three distinct analytical models were implemented:

Model 1: Unadjusted (crude).

Model 2: Adjusted for age, sex, race, education level, marital status, and poverty income ratio (PIR).

Model 3: Further adjusted for smoking status, drinking status, CKM syndrome stage, body mass index (BMI), white blood cell count, and uric acid levels.

To examine potential linear and non-linear relationships between DI-GM scores (including total, beneficial, and unfavorable components) and mortality outcomes, restricted cubic spline (RCS) modeling was implemented with four predetermined knot positions. These knots were strategically positioned at the 5th, 35th, 65th, and 95th percentile values of the DI-GM score distribution [23]. The mediating effect of SII on the association between DI-GM scores and all-cause mortality was investigated through mediation analysis performed with the R software’s “mediation” package. Additional stratified multivariate regression models were employed to conduct subgroup analyses. To ensure robustness of findings, sensitivity analyses were carried out by excluding individuals who experienced mortality events during the initial 24-month follow-up period.

Statistical processing was performed utilizing R software (version 4.4.2), with a predefined threshold of *p* < 0.05 for determining statistical significance.

## 3. Results

### 3.1 Baseline Characteristics

The research involved 7884 American participants aged between 30 and 79 years, showing a median age of 50 (interquartile range: 40–61), with females constituting 52.97% (n = 4182) of the sample (Table 1). The ethnic distribution comprised 7.67% Mexican Americans, 5.49% other Hispanic individuals, 68.87% non-Hispanic Caucasians, 10.61% non-Hispanic African Americans, and 7.36% representing other ethnicities. When analyzing participants based on quartiles of the DI-GM composite score (Table 1), individuals with elevated scores tended to be male, of non-Hispanic White descent, more educated, and with greater household earnings. No notable age variations were found among the groups (*p *= 0.282). Elevated DI-GM scores also correlated with increased alcohol intake, reduced incidence of diabetes, and chronic kidney disease. Nevertheless, no meaningful relationship emerged between DI-GM scores and CKM syndrome progression (*p *= 0.181). Conversely, when examining DI-GM beneficial score quartiles (**Supplementary Table 3**), higher values were connected with decreased occurrence of advanced CKM syndrome stages (Stage 2 and 3, *p *= 0.004). Furthermore, greater DI-GM total scores corresponded with higher body mass index, whereas increased beneficial scores were associated with reduced leukocyte counts and serum uric acid concentrations.

**Table 1.** **Baseline characteristics of participants by DI-GM total score quartiles**.

		Total	Q1 (0, 1, 2)	Q2 (3)	Q3 (4)	Q4 (≥5)	*p*
		N = 7884	N = 2191	N = 1579	N = 1639	N = 2475	
Age, years		50.00 (40.00, 61.00)	51.00 (41.00, 62.00)	50.00 (40.00, 59.00)	50.00 (40.00, 61.00)	51.00 (40.00, 61.00)	0.282
Female		4182 (52.97)	1221 (56.92)	860 (54.77)	858 (52.21)	1243 (49.89)	0.013
Race							<0.001
	Mexican American	1207 (7.67)	290 (7.57)	251 (7.95)	268 (7.86)	398 (7.47)	
	Other Hispanic	891 (5.49)	307 (7.82)	183 (5.81)	173 (4.85)	228 (4.19)	
	Non-Hispanic White	3259 (68.87)	724 (58.16)	613 (66.29)	736 (72.88)	1186 (74.78)	
	Non-Hispanic Black	1594 (10.61)	612 (17.09)	335 (11.81)	272 (8.25)	375 (7.18)	
	Other Race	933 (7.36)	258 (9.35)	197 (8.13)	190 (6.16)	288 (6.38)	
Education							<0.001
	Less than high school	1793 (14.41)	633 (19.28)	375 (14.08)	360 (14.46)	425 (11.38)	
	High school or higher	6087 (85.59)	1557 (80.72)	1202 (85.92)	1279 (85.54)	2049 (88.62)	
Marital status							<0.001
	Not married/cohabiting	2673 (29.77)	860 (34.42)	527 (29.10)	535 (30.47)	751 (26.66)	
	Married/cohabiting	5209 (70.23)	1331 (65.58)	1051 (70.90)	1104 (69.53)	1723 (73.34)	
PIR							<0.001
	<1	1338 (11.66)	478 (17.27)	302 (13.75)	265 (10.42)	293 (7.65)	
	1–3	2918 (33.92)	846 (38.20)	591 (32.30)	593 (34.80)	888 (31.44)	
	≥3	2946 (54.42)	669 (44.53)	564 (53.95)	618 (54.78)	1095 (60.91)	
Drinking		4467 (76.82)	1121 (71.65)	875 (75.44)	971 (77.80)	1500 (80.25)	<0.001
Smoking		3505 (44.32)	972 (44.08)	669 (43.63)	739 (46.62)	1125 (43.44)	0.565
Hypertension		3600 (41.94)	1051 (43.07)	708 (39.69)	734 (41.76)	1107 (42.50)	0.520
Diabetes		1752 (16.85)	514 (18.21)	379 (18.90)	325 (14.59)	534 (16.24)	0.031
Dyslipidemia		5922 (74.25)	1670 (75.58)	1205 (76.65)	1212 (70.84)	1835 (74.19)	0.049
Chronic kidney disease		1147 (12.25)	388 (15.95)	221 (11.36)	228 (11.60)	310 (10.73)	0.001
Antihypertensive medications		2245 (85.07)	652 (87.42)	432 (84.32)	469 (86.22)	692 (83.27)	0.337
Antidiabetic medications		816 (50.22)	219 (49.31)	178 (53.66)	164 (51.35)	255 (48.47)	0.786
Lipid-lowering medications		1292 (75.32)	349 (77.51)	258 (70.20)	292 (78.49)	393 (74.78)	0.183
BMI, kg/m^2^		28.51 (24.88, 33.13)	27.87 (24.34, 32.23)	28.55 (25.04, 33.14)	28.27 (24.73, 32.96)	28.94 (25.27, 33.64)	0.002
Triglycerides, mg/dL		104.00 (72.00, 155.00)	102.00 (69.00, 153.00)	107.00 (72.00, 157.00)	106.00 (72.00, 157.00)	102.00 (73.00, 154.00)	0.328
Total cholesterol, mg/dL		195.00 (171.00, 222.00)	195.00 (169.00, 223.00)	198.00 (172.00, 224.00)	195.00 (172.00, 223.00)	195.00 (170.00, 219.00)	0.122
White blood cells, 1000/µL		6.40 (5.40, 7.80)	6.40 (5.40, 7.80)	6.40 (5.30, 7.80)	6.30 (5.30, 7.80)	6.30 (5.40, 7.70)	0.645
HbA1c, %		5.50 (5.30, 5.80)	5.50 (5.30, 5.80)	5.50 (5.20, 5.80)	5.50 (5.30, 5.80)	5.50 (5.30, 5.80)	0.757
Creatine, mg/dL		0.83 (0.71, 0.97)	0.83 (0.71, 0.99)	0.82 (0.70, 0.96)	0.83 (0.71, 0.96)	0.84 (0.71, 0.97)	0.308
Uric acid, mg/dL		5.40 (4.50, 6.30)	5.50 (4.50, 6.40)	5.40 (4.50, 6.40)	5.40 (4.40, 6.30)	5.40 (4.50, 6.40)	0.920
SII		448.40 (329.00, 633.33)	447.56 (317.69, 651.18)	455.31 (330.06, 630.00)	441.10 (328.50, 624.48)	451.00 (334.53, 635.50)	0.911
DI-GM		2.40 (0.03)	0.91 (0.02)	1.87 (0.03)	2.53 (0.03)	3.58 (0.03)	<0.001
DI-GM_Beneficial		3.86 (0.03)	1.48 (0.02)	3.00 (0.00)	4.00 (0.00)	5.78 (0.02)	<0.001
DI-GM_Unfavorable		2.54 (0.02)	3.43 (0.02)	2.87 (0.03)	2.53 (0.03)	1.80 (0.03)	<0.001
CKM syndrome							0.181
	Stage 0	498 (7.51)	132 (7.39)	97 (7.45)	119 (9.36)	150 (6.49)	
	Stage 1	1480 (21.46)	378 (19.55)	291 (21.53)	304 (19.98)	507 (23.57)	
	Stage 2	5311 (63.68)	1503 (65.36)	1080 (63.64)	1094 (64.34)	1634 (62.19)	
	Stage 3	595 (7.35)	178 (7.69)	111 (7.38)	122 (6.32)	184 (7.74)	
All cause death		469 (4.56)	167 (6.79)	75 (3.08)	103 (5.38)	124 (3.39)	<0.001
Cardiovascular death		105 (1.02)	31 (1.22)	15 (0.68)	31 (1.64)	28 (0.70)	0.015

Abbreviation: BMI, body mass index; CKM, cardiovascular-kidney-metabolic; DI-GM, dietary index for gut microbiota; HbA1c, glycated hemoglobin A1c; PIR, poverty income ratio; SII, systemic inflammation index; NHANES, National Health and Nutrition Examination Survey; IQR, interquartile range. Note: Values are presented as mean (SE) or median (IQR) for continuous variables and number (weighted percentage) for categorical variables. Statistical comparisons were performed using chi-square tests for categorical variables, ANOVA or Kruskal-Wallis tests for continuous variables, as appropriate. Sample sizes for each quartile are provided. Discrepancies in category totals reflect missing or unknown values in NHANES.

### 3.2 Association Between DI-GM and Mortality Outcomes

**Fig. 2.**
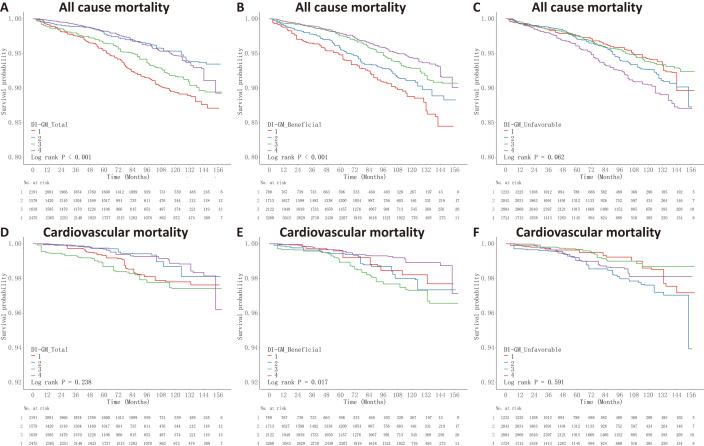
**Kaplan-Meier survival curves for the DI-GM scores and mortality outcomes**. Participants were categorized into quartiles (Q1–Q4) based on the DI-GM total, beneficial, and unfavorable scores, with Q1 representing the lowest and Q4 the highest dietary index scores. Survival probabilities over time are shown according to: (A) DI-GM total score, (B) DI-GM beneficial score, and (C) DI-GM unfavorable score for all-cause mortality; and (D) DI-GM total score, (E) DI-GM beneficial score, and (F) DI-GM unfavorable score for cardiovascular mortality. Log-rank tests were performed to assess differences in survival across quartile groups.

During a median observation period of 77 months, 469 deaths from any cause (4.56%) and 105 cardiovascular-related fatalities (1.02%) were documented. Survival analysis using Kaplan-Meier curves revealed that individuals in the bottom quartiles for both total DI-GM (Q1: scores 0–2) and beneficial component scores (Q1: scores 0–1) exhibited the greatest likelihood of all-cause mortality (log-rank *p* < 0.001, Fig. 2). The association between adverse scores and overall mortality risk was not statistically significant (log-rank *p* = 0.062). Regarding cardiovascular mortality, only the beneficial component demonstrated a meaningful correlation (log-rank *p* = 0.017), with participants in the top quartile (Q4: scores ≥4) displaying the most favorable outcomes.

**Fig. 3.**
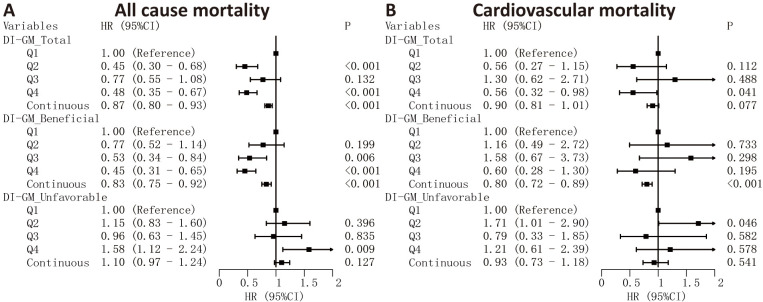
**Cox regression analysis of DI-GM scores and mortality risks**. Associations between DI-GM total, beneficial, and unfavorable scores and mortality outcomes. HRs and 95% CIs are presented for all-cause (A) and cardiovascular (B) mortality. DI-GM scores were analyzed both as categorical variables (quartiles, with Q1 as the reference group) and as continuous variables (per 1-point increase in score).

The results from Cox regression analysis (Fig. 3) demonstrated that every unit increment in the DI-GM total score correlated with a 13% decrease in overall mortality risk (HR = 0.87, 95% CI: 0.80–0.93), while no significant relationship was observed with cardiovascular mortality. Similarly, a one-point rise in the beneficial score corresponded to a 17% reduction in all-cause mortality (HR = 0.83, 95% CI: 0.75–0.92) and a 20% decrease in cardiovascular mortality risk (HR = 0.80, 95% CI: 0.72–0.89). When analyzed by quartiles, participants in the highest quartile of both DI-GM total and beneficial scores exhibited substantially lower all-cause mortality rates compared to the lowest quartile (Q4 vs Q1 HR = 0.48 [95% CI: 0.35–0.67] and 0.45 [95% CI: 0.31–0.65], respectively). Conversely, elevated unfavorable scores were linked to greater all-cause mortality risk (Q4 vs Q1 HR = 1.58, 95% CI: 1.12–2.24), but showed no significant association with cardiovascular mortality.

The findings from the multivariable Cox regression analysis (Model 3) demonstrated that both the overall DI-GM score and its beneficial component exhibited independent correlations with decreased mortality from all causes. Notably, every unit increment in the total score corresponded to a 10% decline in all-cause mortality risk (HR = 0.90, 95% CI: 0.82–0.98), whereas the beneficial component showed an even stronger association with a 12% risk reduction (HR = 0.88, 95% CI: 0.79–0.98; Table 2). In quartile-based comparisons, individuals positioned in the top quartile (Q4) for both the total and beneficial DI-GM scores displayed substantially lower all-cause mortality rates relative to those in the bottom quartile (Q1), with HRs of 0.57 (95% CI: 0.40–0.83) and 0.60 (95% CI: 0.41–0.87), respectively. Regarding cardiovascular mortality outcomes, only the beneficial score maintained statistical significance following comprehensive adjustment (HR = 0.87, 95% CI: 0.77–0.99; Table 3), while the relationship with the total score weakened and lost significance. Importantly, the unfavorable DI-GM score component failed to show any meaningful association with either all-cause or cardiovascular mortality across all adjusted models. When examining quartile distributions, no statistically significant variations in cardiovascular mortality risk were detected between the highest (Q4) and lowest (Q1) quartiles for any DI-GM score components after complete covariate adjustment.

**Table 2.** **Association between DI-GM scores and all-cause mortality**.

		Model 1		Model 2		Model 3	
		HR (95% CI)	*p*-value	HR (95% CI)	*p*-value	HR (95% CI)	*p*-value
DI-GM_Total							
	Q1	1.00 (Reference)		1.00 (Reference)		1.00 (Reference)	
	Q2	0.45 (0.30–0.68)	<0.001	0.52 (0.34–0.80)	0.003	0.53 (0.33–0.85)	0.008
	Q3	0.77 (0.55–1.08)	0.132	0.80 (0.56–1.14)	0.224	0.73 (0.50–1.07)	0.104
	Q4	0.48 (0.35–0.67)	<0.001	0.57 (0.41–0.78)	<0.001	0.57 (0.40–0.83)	0.003
*p* for trend			<0.001		0.001		0.001
Continuous		0.87 (0.80–0.93)	<0.001	0.90 (0.83–0.97)	0.005	0.90 (0.82–0.98)	0.010
DI-GM_Beneficial							
	Q1	1.00 (Reference)		1.00 (Reference)		1.00 (Reference)	
	Q2	0.77 (0.52–1.14)	0.199	0.96 (0.64–1.44)	0.829	0.94 (0.60–1.49)	0.803
	Q3	0.53 (0.34–0.84)	0.006	0.67 (0.44–1.04)	0.077	0.67 (0.41–1.07)	0.096
	Q4	0.45 (0.31–0.65)	<0.001	0.58 (0.41–0.82)	0.002	0.60 (0.41–0.87)	0.008
*p* for trend			<0.001		<0.001		0.002
Continuous		0.83 (0.75–0.92)	<0.001	0.87 (0.79–0.95)	0.003	0.88 (0.79–0.98)	0.019
DI-GM_Unfavorable							
	Q1	1.00 (Reference)		1.00 (Reference)		1.00 (Reference)	
	Q2	1.15 (0.83–1.60)	0.396	1.13 (0.81–1.58)	0.476	1.09 (0.77–1.54)	0.641
	Q3	0.96 (0.63–1.45)	0.835	0.97 (0.64–1.47)	0.870	0.94 (0.59–1.48)	0.777
	Q4	1.58 (1.12–2.24)	0.009	1.35 (0.94–1.93)	0.099	1.46 (0.97–2.19)	0.069
*p* for trend			0.059		0.237		0.158
Continuous		1.10 (0.97–1.24)	0.127	1.06 (0.94–1.20)	0.320	1.09 (0.95–1.26)	0.217

Model 1: Unadjusted (crude model). Model 2: Adjusted for age, sex, race, education level, marital status, and PIR. Model 3: Adjusted for age, sex, race, education level, marital status, PIR, drinking status, smoking status, CKM syndrome stages, BMI, white blood cell count, and uric acid level. HR and 95% CI for all-cause mortality across quartiles of DI-GM total, beneficial, and unfavorable scores (Q1 as reference), and per 1-point increase in score (continuous).

**Table 3.** **Association between DI-GM scores and cardiovascular mortality**.

		Model 1		Model 2		Model 3	
		HR (95% CI)	*p*-value	HR (95% CI)	*p*-value	HR (95% CI)	*p*-value
DI-GM_Total							
	Q1	1.00 (Reference)		1.00 (Reference)		1.00 (Reference)	
	Q2	0.56 (0.27–1.15)	0.112	0.68 (0.33–1.39)	0.291	0.63 (0.29–1.40)	0.260
	Q3	1.30 (0.62–2.71)	0.488	1.46 (0.68–3.15)	0.329	1.13 (0.50–2.53)	0.770
	Q4	0.56 (0.32–0.98)	0.041	0.73 (0.39–1.37)	0.323	0.73 (0.37–1.45)	0.366
*p* for trend			0.227		0.714		0.635
Continuous		0.90 (0.81–1.01)	0.077	0.96 (0.84–1.09)	0.503	0.95 (0.82–1.10)	0.506
DI-GM_Beneficial							
	Q1	1.00 (Reference)		1.00 (Reference)		1.00 (Reference)	
	Q2	1.16 (0.49–2.72)	0.733	1.35 (0.61–3.01)	0.463	1.47 (0.58–3.68)	0.415
	Q3	1.58 (0.67–3.73)	0.298	1.96 (0.85–4.50)	0.114	2.05 (0.84–4.96)	0.113
	Q4	0.60 (0.28–1.30)	0.195	0.78 (0.37–1.64)	0.509	0.93 (0.41–2.12)	0.872
*p* for trend			0.009		0.110		0.329
Continuous		0.80 (0.72–0.89)	<0.001	0.84 (0.75–0.94)	0.003	0.87 (0.77–0.99)	0.030
DI-GM_Unfavorable							
	Q1	1.00 (Reference)		1.00 (Reference)		1.00 (Reference)	
	Q2	1.71 (1.01–2.90)	0.046	1.59 (0.89–2.83)	0.117	1.44 (0.92–2.26)	0.115
	Q3	0.79 (0.33–1.85)	0.582	0.71 (0.29–1.72)	0.448	0.74 (0.32–1.73)	0.494
	Q4	1.21 (0.61–2.39)	0.578	0.87 (0.41–1.84)	0.712	1.05 (0.47–2.34)	0.905
*p* for trend			0.589		0.225		0.602
Continuous		0.93 (0.73–1.18)	0.541	0.85 (0.66–1.09)	0.203	0.91 (0.68–1.21)	0.527

Model 1: Unadjusted (crude model). Model 2: Adjusted for age, sex, race, education level, marital status, and PIR. Model 3: Adjusted for age, sex, race, education level, marital status, PIR, drinking status, smoking status, CKM syndrome stages, BMI, white blood cell count, and uric acid level. HR and 95% CI for cardiovascular mortality across quartiles of DI-GM total, beneficial, and unfavorable scores (Q1 as reference), and per 1-point increase in score (continuous).

**Fig. 4.**
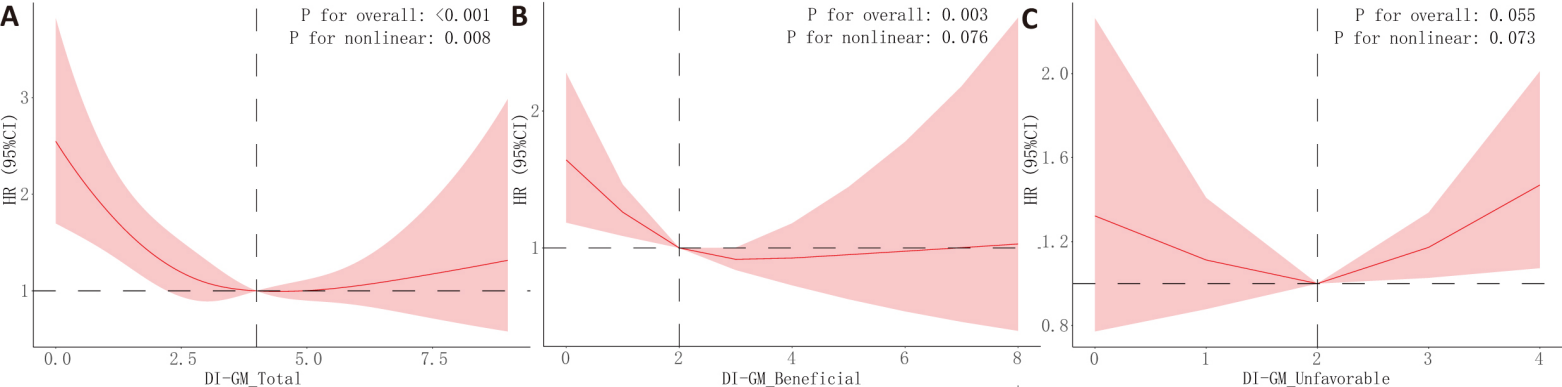
**Restricted cubic spline (RCS) analysis results for the association between DI-GM scores and all-cause mortality**. The associations between DI-GM scores and all-cause mortality outcomes were analyzed using RCS models, adjusted for covariates in Model 3. The relationships between DI-GM total score (A), beneficial score (B), unfavorable score (C) and all-cause mortality were evaluated. The figure displays the HR (solid lines) with 95% CI (shaded areas).

RCS (Fig. 4) revealed a U-shaped relationship between DI-GM total score and all-cause mortality (*p* for non-linearity = 0.008), a linear association for beneficial scores (*p* for non-linearity = 0.076), and no significant pattern for unfavorable scores. For cardiovascular mortality (**Supplementary Fig. 1**), the DI-GM beneficial score showed an inverted U-shaped association (*p* for non-linearity = 0.031), while total and unfavorable scores showed no significant trends.

### 3.3 Mediation Analysis

**Fig. 5.**
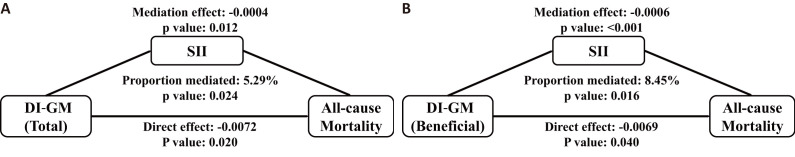
**Mediation analysis examining the role of SII in the relationship between DI-GM scores and mortality from all causes**. The study performed mediation analyses to assess whether systemic inflammation index (SII) mediated the link between DI-GM composite score (A), positive component score (B) and overall mortality. These analyses incorporated adjustments for the same covariates used in Model 3, with the exception of leukocyte count.

The overall and favorable DI-GM scores showed inverse correlations with SII, demonstrating β values of –7.35 (95% CI: –12.46 to –2.24) and –12.86 (95% CI: –19.64 to –6.07), respectively (Table 4). Mediation assessments indicated that SII accounted for 5.29% (95% CI: 1.44%–10%, *p *= 0.024) of the total DI-GM score’s impact and 8.45% (95% CI: 2.12%–18%, *p* = 0.016) of the beneficial DI-GM score’s influence on mortality from all causes (Fig. 5). These findings imply that diets promoting gut microbiota health might lower mortality risk in part by decreasing systemic inflammatory responses.

**Table 4.** **Linear regression analysis between DI-GM scores and SII**.

	β (95% CI)	*p*-value
DI-GM_Total	–7.35 (–12.46 to –2.24)	0.006
DI-GM_Beneficial	–12.86 (–19.64 to –6.07)	<0.001
DI-GM_Unfavorable	–0.44 (–10.69 to 9.81)	0.933

**Note:** β coefficients and 95% CIs represent the estimated change in systemic inflammation index (SII) per 1-point increase in each DI-GM score. Analyses were adjusted for covariates included in model 3.

### 3.4 Subgroup and Sensitivity Analysis

Analyses conducted across various demographic subgroups, including age, gender, ethnicity, body mass index, and stages of CKM syndrome, demonstrated stable relationships between overall DI-GM scores or favorable dietary indices and mortality risks (all interaction *p*-values exceeded 0.05; refer to Table 5 and **Supplementary Table 4**). When participants who died within the initial 24 months of follow-up were excluded from consideration, the beneficial DI-GM score maintained its significant correlation with cardiovascular-related deaths (multivariable-adjusted HR = 0.87, 95% confidence interval: 0.77–0.99; see **Supplementary Table 5**), while its association with overall mortality showed reduced significance (fully adjusted HR = 0.92, 95% CI: 0.81–1.03; **Supplementary Table 5**).

**Table 5.** **Subgroup analysis of adjusted HR for mortality outcomes by DI-GM beneficial scores**.

Subgroup		All-cause mortality			Cardiovascular mortality		
		HR (95% CI)	*p*-value	*p* for interaction	HR (95% CI)	*p*-value	*p* for interaction
Age, years				0.768			0.643
	30–45	0.83 (0.59–1.16)	0.265		1.02 (0.66–1.56)	0.944	
	45–60	0.89 (0.68–1.17)	0.407		0.73 (0.53–1.01)	0.060	
	≥60	0.89 (0.80–0.99)	0.035		0.86 (0.75–0.99)	0.038	
Sex				0.669			0.839
	Male	0.90 (0.79–1.03)	0.139		0.83 (0.71–0.97)	0.019	
	Female	0.88 (0.77–1.00)	0.057		0.91 (0.73–1.14)	0.403	
Race				0.483			0.225
	Mexican American	0.93 (0.68–1.28)	0.667		1.02 (0.58–1.79)	0.938	
	Other Hispanic	1.21 (0.90–1.62)	0.213		1.38 (0.89–2.15)	0.151	
	Non-Hispanic White	0.86 (0.77–0.95)	0.005		0.80 (0.69–0.92)	0.002	
	Non-Hispanic Black	0.91 (0.76–1.09)	0.319		0.98 (0.75–1.29)	0.888	
	Other Race	0.95 (0.70–1.29)	0.741		0.95 (0.57–1.59)	0.858	
BMI				0.557			0.804
	<25	0.83 (0.66–1.04)	0.112		0.95 (0.78–1.16)	0.634	
	25–30	0.93 (0.79–1.10)	0.417		0.88 (0.66–1.16)	0.354	
	≥30	0.87 (0.76–1.00)	0.049		0.81 (0.64–1.02)	0.078	
CKM syndrome				0.747			0.396
	Stage 0	0.30 (0.20–0.45)	<0.001		0.16 (0.08–0.32)	<0.001	
	Stage 1	0.91 (0.70–1.17)	0.449		0.72 (0.42–1.24)	0.241	
	Stage 2	0.88 (0.80–0.98)	0.018		0.83 (0.74–0.93)	<0.001	
	Stage 3	0.97 (0.79–1.19)	0.749		1.06 (0.64–1.75)	0.820	

HRs accompanied by 95% CIs for both all-cause and cardiovascular mortality are displayed across various subgroups (including age, gender, ethnicity, body mass index, and stages of CKM syndrome), with the DI-GM beneficial score analyzed as a continuous parameter. These evaluations were modified to account for the confounding factors incorporated in Model 3.

## 4. Discussion

This research investigated the association between DI-GM scores (including overall, favorable, and adverse components) and mortality rates, utilizing NHANES data collected from 2007 to 2018. The results demonstrate that dietary patterns promoting beneficial gut microbiota are correlated with decreased risks of both all-cause and cardiovascular-related deaths in patients with CKM syndrome across stages 0 to 3. Notably, every unit increment in the favorable DI-GM component corresponded to a 12% decrease in overall mortality and a 13% decline in cardiovascular-related deaths. The analysis revealed a U-shaped curve for the relationship between total DI-GM scores and all-cause mortality, whereas the beneficial component showed a direct linear correlation. Furthermore, part of this effect may be mediated through reduced systemic inflammation. Notably, sensitivity analyses confirmed that beneficial dietary components had a more consistent association with cardiovascular mortality than with all-cause mortality. The findings indicate that nutritional interventions could contribute to enhancing the prognosis of patients diagnosed with initial-phase CKM syndrome over extended periods.

Extensive research has confirmed the strong association between metabolic disorders and elevated risks of cardiovascular and renal disease occurrence and fatality [24, 25, 26]. The Global Burden of Disease analysis identifies poorly managed blood sugar levels and excessive body weight as key factors in the development of coronary artery disease, particularly in areas with limited healthcare resources [24, 27]. The concept of CKM syndrome emphasizes the complex interplay among metabolic, cardiac, and kidney functions, demonstrating the necessity for comprehensive management strategies. The rising incidence of this condition across the United States in recent years represents a substantial healthcare challenge [3, 5]. Multiple pathophysiological processes, such as persistent inflammatory responses, oxidative damage, diminished insulin sensitivity, lipid-induced cellular injury, and disrupted metabolic pathways, exacerbate clinical outcomes, especially in patients with severe CKM syndrome manifestations [28]. This complexity presents challenges in identifying effective interventions, especially during the earlier stages of the syndrome.

Dietary intake plays a critical role in regulating these pathways. Evidence increasingly supports the influence of diet on health outcomes through mechanisms mediated by the gut microbiota [12, 13, 14]. While the association between dietary habits and various illnesses, including stroke, diabetes, malignancies, and cardiovascular disorders, has been extensively documented, its impact on the clinical outcomes of CKM syndrome has not been sufficiently investigated.

Emerging research has highlighted the crucial involvement of intestinal microbial communities in the pathogenesis and advancement of various diseases. These microbial populations generate bioactive compounds that modulate inflammatory pathways, lipid homeostasis, and insulin sensitivity [29, 30, 31, 32]. Although scientific attention has focused on the microbiota’s impact on disease initiation and progression, its potential link with mortality outcomes, especially in cases of CKM syndrome, remains relatively understudied.

The intestinal microbiome exerts direct effects on cardiovascular health via bioactive metabolites, including trimethylamine-N-oxide, short-chain fatty acids, and bile acids, compounds that contribute to atherosclerotic development and elevated cardiovascular risk [29, 33]. Considering these extensive physiological effects, microbial communities in the gut have emerged as promising intervention targets for cardiovascular disease management, with potential benefits extending to mortality reduction [34]. Our research aligns with this perspective, revealing that elevated DI-GM scores—especially those indicative of favorable dietary constituents—correlate with reduced cardiovascular-related deaths. These observations highlight the significance of nutritional approaches that support intestinal microbial balance and their capacity to enhance cardiovascular prognosis and lifespan among patients in the initial phases of CKM syndrome [35].

Notably, the DI-GM demonstrated a curvilinear relationship with overall mortality rates, indicating that consuming extremely high quantities of foods beneficial to gut microbiota might not yield further advantages and could potentially be detrimental. This pattern of non-linear correlations has been previously documented in research investigating the consumption of proteins, carbohydrates, and fruits concerning mortality from all causes and cardiovascular diseases, highlighting the critical need for maintaining dietary equilibrium [36, 37]. One possible explanation is a secondary imbalance in gut microbiota composition, which warrants further investigation.

Moreover, evidence suggests that chronic systemic inflammation serves as a key pathway linking DI-GM with increased mortality risks. The Dietary Inflammatory Index (DII), a validated tool for assessing the inflammatory properties of food components, quantifies their influence on biomarkers including C-reactive protein, tumor necrosis factor, and various interleukins [13]. Analysis of NHANES 1999–2018 datasets revealed significant correlations between elevated DII scores and heightened risks of all-cause mortality (HR = 1.06, 95% CI: 1.02–1.11) as well as cardiovascular-related deaths (HR = 1.08, 95% CI: 1.01–1.15) in patients diagnosed with cardiovascular disease [38]. These detrimental outcomes are primarily attributed to inflammatory cytokine-induced processes such as impaired endothelial function, enhanced monocyte migration, foam cell development, and excessive lipid deposition [39, 40].

Although DI-GM and DII exhibit distinct compositional elements and scoring approaches, our research demonstrated that dietary patterns promoting gut microbiota health correlated with reduced systemic inflammatory markers. Mediation analysis outcomes additionally indicate that the mortality-protective mechanism of DI-GM could be partly attributed to its inflammation-reducing characteristics. The potential pathways through which gut microbiota affects systemic inflammation may involve bacterial translocation processes and immunoregulatory metabolites [41, 42].

In sensitivity assessments that eliminated subjects deceased during the initial 24-month follow-up period, the relationship between DI-GM beneficial scores and all-cause mortality lost statistical significance. However, the inverse relationship with cardiovascular mortality remained consistent. This finding indicates that the initial association with all-cause mortality may have been partly influenced by underlying health conditions that affected both dietary behaviors and short-term mortality risk. In contrast, the persistent association with cardiovascular mortality suggests a more independent and long-term protective effect of gut microbiota-beneficial dietary patterns, particularly given the chronic nature and gradual progression of cardiovascular disease.

### Strengths and Limitations

The research underscores the significant influence of dietary habits on mortality outcomes, particularly for patients in CKM syndrome stages 0–3, a population group where dietary intervention studies remain scarce. Utilizing comprehensive NHANES data enhances the validity and applicability of these findings across diverse demographic groups in the United States. Furthermore, through distinct evaluation of both positive and negative elements within the DI-GM scoring system, our analysis indicates that consuming gut microbiota-friendly foods may serve as a key factor in lowering mortality rates, potentially mediated by their impact on inflammatory responses throughout the body.

Several important limitations warrant consideration in this study. Firstly, the cross-sectional nature of the research design restricts our capacity to establish causal relationships between DI-GM scores and mortality results. Future investigations employing longitudinal approaches would be essential to verify these observed connections and assess the sustained impacts of dietary habits. Secondly, the nutritional data collection relied on participant-reported 24-hour dietary recalls, a methodology potentially vulnerable to memory biases and incomplete reporting, which might compromise the precision of DI-GM computations. Thirdly, despite comprehensive adjustments for numerous sociodemographic characteristics, lifestyle variables, and clinical parameters, the possibility of unmeasured confounding factors cannot be entirely ruled out. In particular, physical activity, total energy intake, and medication use (antihypertensive, antidiabetic, and lipid-lowering drugs) were not incorporated into the models due to a large proportion of missing data, which may have limited our ability to fully account for these influences. Fourth, although NHANES data are nationally representative, findings may not be directly applicable to populations outside the US due to differences in dietary behaviors, cultural practices, and genetic backgrounds. Finally, as with all survival analyses, censoring could have affected the precision of the survival estimates. However, since censoring patterns were comparable across quartiles, the likelihood of systematic bias is minimized.

## 5. Conclusions

Elevated DI-GM scores, which indicate increased consumption of foods beneficial to gut microbiota, correlate with reduced risks of mortality from all causes and cardiovascular diseases among individuals in CKM syndrome stages 0–3. The association is particularly strong for cardiovascular-related deaths. Mediation analyses additionally indicate that systemic inflammation may serve as a mediating pathway. These results imply that dietary interventions aimed at enhancing gut microbiota health could represent a viable approach for improving long-term prognosis in this patient group. Further investigation through longitudinal cohort studies and clinical trials is necessary to confirm these findings and inform dietary guidelines based on robust evidence.

## Availability of Data and Materials

Data of NHANES is available from the NHANES website (https://www.cdc.gov/nchs/nhanes/). And the data used for analysis in this study is available from the corresponding author on reasonable request.

## Supplementary Material

Supplementary material associated with this article can be found, in the online version, at https://doi.org/10.31083/RCM45493.
