# Supplementary material for: Dietary Index for Gut Microbiota and Risk of All-Cause and Cardiovascular Mortality Across Cardiovascular-Kidney-Metabolic Syndrome Stages 0–3: A Nationwide Prospective Cohort Study
Source: Rev Cardiovasc Med. 2026 Feb 13;27(2):45493. doi: 10.31083/RCM45493 (PMC12959983; doi:10.31083/RCM45493)
Supplement: Supplementary file 1 [file 2153-8174-27-2-45493-s1.docx]

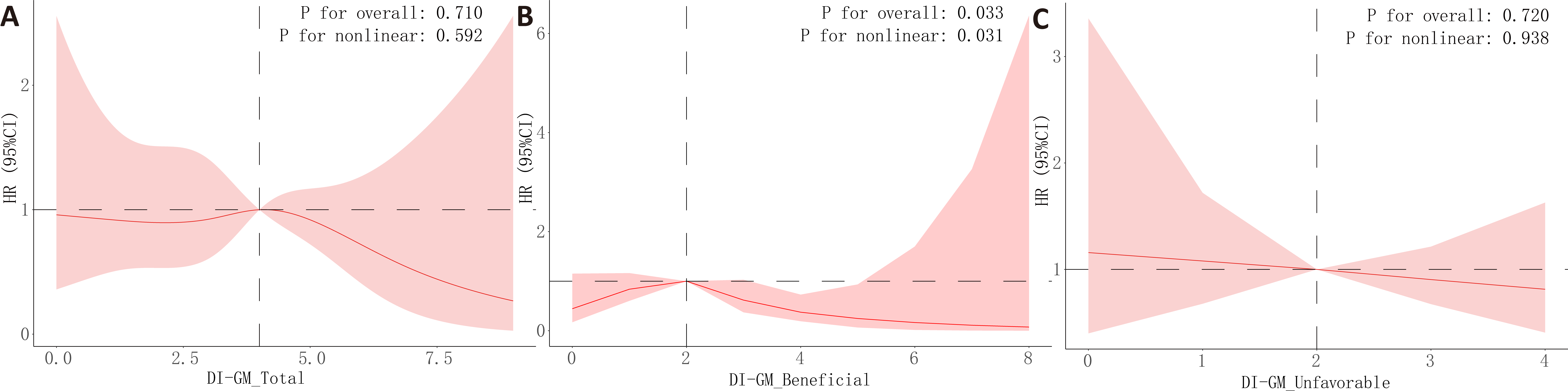


**Supplementary Fig. 1.** RCS analysis results for the association between DI-GM scores and cardiovascular mortality

The associations between DI-GM scores and cardiovascular mortality outcomes were analyzed using RCS models, adjusted for covariates in Model 3. The relationships between DI-GM total score (A), beneficial score (B), unfavorable score (C) and all-cause mortality were evaluated. The figure displays the HR (solid lines) with 95% CI (shaded areas).

**Supplementary Table 1.** Definition of Cardiovascular-Kidney-Metabolic Syndrome Stages

| CKM syndrome stage definition criteria | |
| --- | --- |
| **Stage 0** | Individuals with normal BMI (18.5-24.9 kg/m^2) and waist circumference (<88cm for woman and <102 cm for man), normal blood glucose, blood pressure, and lipid levels, with no evidence of CKD or CVD, and an estimated 10-year CVD risk <20%. |
| **Stage 1** | Individuals with overweight/obesity (≥25 kg/m^2), central adiposity (waist circumference ≥88cm for woman and ≥102 cm for man), or pre-diabetes (FBG 100-124mg/dL, HbA1c 5.7-6.4%), but without other metabolic abnormalities or CKD, and with an estimated 10-year CVD risk <20%. |
| **Stage 2** | Individuals with at least one metabolic abnormality (hypertension, diabetes, hypertriglyceridemia, metabolic syndrome) or with CKD, and an estimated 10-year CVD risk <20%. |
| **Stage 3** | Individuals with at least one metabolic abnormality (hypertension, diabetes, hypertriglyceridemia, or metabolic syndrome) or with CKD, and an estimated 10-year CVD risk ≥20%. |
| **Stage 4** | Individuals with established clinical CVD, including myocardial infarction, heart failure, angina, coronary heart disease, or stroke. |

**Abbreviations:** BMI, body mass index; CKM, cardiovascular-kidney-metabolic; CKD, chronic kidney disease; CVD, cardiovascular disease; FBG: fasting blood glucose; HbA1c: hemoglobin A1c.

**Note:** 10-year CVD risk was estimated using the PREVENT equation.

**Supplementary Table 2.** Scoring criteria for dietary index for gut microbiota

| **Scoring** | **Component** | **Food** |
| --- | --- | --- |
| **Beneficial to gut microbiota:**  For each component, a score of 1 if consumption at or above the sex-specific median, else 0. | Avocados | Avocados |
|  | Broccoli | Broccoli |
|  | Chickpea | Chickpea |
|  | Coffee | Coffee |
|  | Cranberries | Cranberries |
|  | Fermented dairy | Yogurt, cheese, kefir, sour cream, buttermilk |
|  | Fiber |  |
|  | Green tea | Green tea |
|  | Soybean | Soy milk, Tofu |
|  | Whole grains | Whole grains, containing the entire grain kernel. |
| **Unfavorable to gut microbiota:**  For each remaining component, a score of 0 if consumption at or above the sex-specific median, else 1. | Processed meat | Frankfurters, sausages, corned beef, and luncheon meat. |
|  | Red meat | Beef, veal, pork, lamb, and game meat. |
|  | Refined grains | Refined grains that do not contain all of the components of the entire grain kernel |
|  | High-fat diet (% energy) | 0 if consumption at or above 40% energy from fat, else 1. |

**Supplementary Table 3.** Baseline characteristics of participants by DI-GM beneficial score quartiles

|  | **Total**  **N=7884** | **Q1 (0, 1)**  **N=789** | **Q2 (2)**  **N=1713** | **Q3 (3)**  **N=2122** | **Q4 (≥4)**  **N=3260** | **P** |
| --- | --- | --- | --- | --- | --- | --- |
| **Age, years** | 50.00 (40.00, 61.00) | 51.00 (40.00, 61.00) | 50.00 (40.00, 60.00) | 50.00 (40.00, 60.00) | 51.00 (40.00, 61.00) | 0.122 |
| **Female** | 4182 (52.97) | 433 (58.87) | 907 (53.07) | 1118 (51.29) | 1724 (52.87) | 0.108 |
| **Race** |  |  |  |  |  | <0.001 |
| Mexican American | 1207 (7.67) | 98 (7.49) | 257 (8.01) | 365 (8.84) | 487 (6.88) |  |
| Other Hispanic | 891 (5.49) | 98 (6.98) | 237 (7.56) | 235 (5.39) | 321 (4.42) |  |
| Non-Hispanic White | 3259 (68.87) | 244 (54.83) | 613 (61.77) | 871 (68.21) | 1531 (74.70) |  |
| Non-Hispanic Black | 1594 (10.61) | 269 (21.71) | 434 (15.02) | 408 (10.09) | 483 (7.11) |  |
| Other Race | 933 (7.36) | 80 (9.00) | 172 (7.64) | 243 (7.47) | 438 (6.89) |  |
| **Education** |  |  |  |  |  | <0.001 |
| Less than high school | 1793 (14.41) | 246 (22.34) | 504 (19.45) | 476 (14.02) | 567 (11.13) |  |
| High school or higher | 6087 (85.59) | 543 (77.66) | 1208 (80.55) | 1645 (85.98) | 2691 (88.87) |  |
| **Marital status** |  |  |  |  |  | <0.001 |
| Not married/cohabiting | 2673 (29.77) | 318 (35.83) | 642 (33.32) | 728 (30.36) | 985 (26.87) |  |
| Married/cohabiting | 5209 (70.23) | 471 (64.17) | 1071 (66.68) | 1394 (69.64) | 2273 (73.13) |  |
| **PIR** |  |  |  |  |  | <0.001 |
| <1 | 1338 (11.66) | 171 (17.97) | 381 (17.22) | 395 (12.71) | 391 (7.62) |  |
| 1-3 | 2918 (33.92) | 333 (44.55) | 667 (36.03) | 817 (35.75) | 1101 (30.13) |  |
| ≥3 | 2946 (54.42) | 208 (37.48) | 509 (46.76) | 748 (51.53) | 1481 (62.24) |  |
| **Drinking** | 4467 (76.82) | 405 (68.58) | 928 (75.46) | 1164 (75.64) | 1970 (79.45) | <0.001 |
| **Smoking** | 3505 (44.32) | 363 (42.74) | 792 (46.67) | 940 (45.50) | 1410 (42.93) | 0.222 |
| **Hypertension** | 3600 (41.94) | 413 (45.74) | 800 (42.87) | 945 (41.55) | 1442 (41.10) | 0.299 |
| **Diabetes** | 1752 (16.85) | 214 (21.62) | 399 (18.04) | 469 (17.12) | 670 (15.35) | 0.014 |
| **Dyslipidemia** | 5922 (74.25) | 612 (77.89) | 1305 (76.19) | 1609 (74.57) | 2396 (72.61) | 0.086 |
| **Antihypertensive medications** | 2245 (85.07) | 757 (86.52) | 569 (85.46) | 494 (82.96) | 425 (85.09) | 0.622 |
| **Antidiabetic medications** | 816 (50.22) | 284 (53.91) | 210 (50.63) | 172 (50.38) | 150 (45.04) | 0.314 |
| **Lipid-lowering medications** | 1292 (75.32) | 417 (77.37) | 315 (68.07) | 292 (83.00) | 268 (73.27) | <0.001 |
| **Chronic Kidney Disease** | 1147 (12.25) | 163 (19.16) | 276 (14.25) | 286 (10.75) | 422 (11.09) | <0.001 |
| **BMI, kg/m^2** | 28.51 (24.88, 33.13) | 28.99 (24.98, 33.16) | 28.40 (25.07, 33.04) | 28.76 (25.03, 33.52) | 28.40 (24.67, 32.95) | 0.159 |
| **Triglycerides, mg/dL** | 104.00 (72.00, 155.00) | 108.00 (75.00, 155.00) | 104.00 (71.00, 156.00) | 108.00 (73.00, 160.00) | 101.00 (71.00, 151.00) | 0.173 |
| **Total Cholesterol, mg/dL** | 195.00 (171.00, 222.00) | 195.00 (172.00, 223.00) | 194.00 (167.00, 221.00) | 197.00 (172.00, 226.00) | 195.00 (171.00, 220.00) | 0.051 |
| **White Blood Cells, 1000/μL** | 6.40 (5.40, 7.80) | 6.60 (5.40, 8.00) | 6.60 (5.50, 8.00) | 6.40 (5.40, 7.90) | 6.20 (5.30, 7.60) | <0.001 |
| **HbA1c, %** | 5.50 (5.30, 5.80) | 5.50 (5.30, 5.90) | 5.50 (5.20, 5.80) | 5.50 (5.30, 5.80) | 5.50 (5.20, 5.80) | 0.015 |
| **Creatine, mg/dL** | 0.83 (0.71, 0.97) | 0.84 (0.72, 1.00) | 0.84 (0.72, 1.00) | 0.82 (0.71, 0.96) | 0.84 (0.71, 0.97) | 0.234 |
| **Uric Acid, mg/dL** | 5.40 (4.50, 6.30) | 5.50 (4.50, 6.40) | 5.40 (4.40, 6.40) | 5.40 (4.60, 6.40) | 5.40 (4.40, 6.30) | 0.047 |
| **SII** | 448.40 (329.00, 633.33) | 439.20 (316.80, 638.40) | 462.00 (331.29, 654.50) | 458.89 (336.54, 652.82) | 440.64 (323.81, 618.10) | 0.076 |
| **DI-GM** | 2.40 (0.03) | 0.00 (0.00) | 1.00 (0.00) | 2.00 (0.00) | 3.64 (0.02) | <0.001 |
| **DI-GM_Beneficial** | 3.86 (0.03) | 1.23 (0.06) | 2.33 (0.03) | 3.52 (0.03) | 5.16 (0.03) | <0.001 |
| **DI-GM_Unfavorable** | 2.54 (0.02) | 2.77 (0.06) | 2.67 (0.03) | 2.48 (0.03) | 2.48 (0.02) | <0.001 |
| **CKM Syndrome** |  |  |  |  |  | 0.004 |
| Stage 0 | 498 (7.51) | 40 (5.39) | 93 (6.53) | 140 (7.60) | 225 (8.25) |  |
| Stage 1 | 1480 (21.46) | 121 (16.57) | 281 (19.07) | 404 (21.05) | 674 (23.56) |  |
| Stage 2 | 5311 (63.68) | 566 (69.88) | 1191 (66.45) | 1432 (64.93) | 2122 (60.70) |  |
| Stage 3 | 595 (7.35) | 62 (8.16) | 148 (7.95) | 146 (6.42) | 239 (7.50) |  |
| **All cause death** | 469 (4.56) | 72 (7.89) | 114 (5.86) | 123 (4.17) | 160 (3.66) | <0.001 |
| **Cardiovascular death** | 105 (1.02) | 11 (1.03) | 22 (1.14) | 38 (1.60) | 34 (0.64) | 0.020 |

**Abbreviation:** BMI: body mass index. CKM: Cardiovascular-Kidney-Metabolic. DI-GM: dietary index for gut microbiota. HbA1c: glycated hemoglobin A1c. PIR: poverty income ratio. SII: systemic inflammation index.

**Note:** Values are presented as mean (SE) or median (IQR) for continuous variables and number (weighted percentage) for categorical variables. Statistical comparisons were performed using chi-square tests for categorical variables, ANOVA or Kruskal-Wallis tests for continuous variables, as appropriate. Sample sizes for each quartile are provided. Discrepancies in category totals reflect missing or unknown values in NHANES.

**Supplementary Table 4.** Subgroup analysis of adjusted HR for mortality outcomes by DI-GM total scores

| **Subgroup** | **All-cause mortality** | | | **Cardiovascular mortality** | | |  |
| --- | --- | --- | --- | --- | --- | --- | --- |
|  | **HR (95% CI)** | **P** | **P for interaction** | **HR (95% CI)** | **P** | **P for interaction** |  |
| **Age, years** |  |  | 0.356 |  |  | 0.973 |  |
| 30-45 | 0.84 (0.67-1.06) | 0.143 |  | 1.09 (0.76-1.56) | 0.650 |  |  |
| 45-60 | 0.87 (0.72-1.06) | 0.157 |  | 0.93 (0.65-1.32) | 0.672 |  |  |
| ≥60 | 0.94 (0.87-1.02) | 0.145 |  | 0.97 (0.85-1.10) | 0.595 |  |  |
| **Sex** |  |  | 0.910 |  |  | 0.208 |  |
| Male | 0.91 (0.83-1.01) | 0.085 |  | 0.89 (0.75-1.06) | 0.193 |  |  |
| Female | 0.92 (0.82-1.02) | 0.109 |  | 1.18 (1.02-1.36) | 0.022 |  |  |
| **Race** |  |  | 0.195 |  |  | 0.184 |  |
| Mexican American | 0.88 (0.71-1.08) | 0.223 |  | 0.88 (0.64-1.22) | 0.449 |  |  |
| Other Hispanic | 1.22 (0.97-1.53) | 0.086 |  | 1.44 (0.81-2.53) | 0.211 |  |  |
| Non-Hispanic White | 0.88 (0.81-0.95) | 0.002 |  | 0.92 (0.79-1.08) | 0.317 |  |  |
| Non-Hispanic Black | 0.98 (0.82-1.17) | 0.848 |  | 1.21 (0.93-1.58) | 0.153 |  |  |
| Other Race | 1.02 (0.78-1.33) | 0.903 |  | 0.85 (0.56-1.28) | 0.441 |  |  |
| **BMI** |  |  | 0.607 |  |  | 0.609 |  |
| <25 | 0.84 (0.72-0.98) | 0.028 |  | 0.97 (0.74-1.28) | 0.855 |  |  |
| 25-30 | 0.91 (0.79-1.05) | 0.211 |  | 0.88 (0.69-1.13) | 0.321 |  |  |
| ≥30 | 0.93 (0.82-1.05) | 0.235 |  | 0.98 (0.79-1.23) | 0.885 |  |  |
| **CKM syndrome** |  |  | 0.952 |  |  | 0.373 |  |
| Stage 0 | 0.44 (0.29-0.67) | <0.001 |  | 8.31 (2.55-27.1) | <0.001 |  |  |
| Stage 1 | 0.90 (0.72-1.12) | 0.357 |  | 1.01 (0.70-1.45) | 0.966 |  |  |
| Stage 2 | 0.90 (0.84-0.98) | 0.010 |  | 0.92 (0.81-1.05) | 0.207 |  |  |
| Stage 3 | 0.99 (0.83-1.18) | 0.940 |  | 1.26 (0.76-2.08) | 0.369 |  |  |

HRs with 95% CIs for all-cause and cardiovascular mortality are presented by subgroup (age, sex, race, BMI, CKM syndrome stages), with DI-GM total score modeled as a continuous variable. Analyses were adjusted for covariates included in Model 3

**Supplementary Table 5.** Sensitivity Analysis of the Association Between DI-GM Scores and Mortality after Excluding Deaths within First Two Years of Follow-Up

|  | **Model 1** | | **Model 2** | | **Model 3** | |
| --- | --- | --- | --- | --- | --- | --- |
|  | **HR (95% CI)** | **P value** | **HR (95% CI)** | **P value** | **HR (95% CI)** | **P value** |
| **All-cause mortality** | | | | | | |
| **DI-GM_Total** | 0.89 (0.81-0.98) | 0.015 | 0.93 (0.85-1.02) | 0.128 | 0.93 (0.84-1.02) | 0.13 |
| **DI-GM_Beneficial** | 0.88 (0.79-0.99) | 0.031 | 0.91 (0.82-1.01) | 0.088 | 0.92 (0.81-1.03) | 0.148 |
| **DI-GM_Unfavorable** | 1.12 (0.98-1.28) | 0.099 | 1.04 (0.90-1.20) | 0.587 | 1.06 (0.91-1.24) | 0.442 |
| **Cardiovascular mortality** | | | | | | |
| **DI-GM_Total** | 0.89 (0.77-1.01) | 0.077 | 0.95 (0.81-1.10) | 0.473 | 0.94 (0.80-1.10) | 0.45 |
| **DI-GM_Beneficial** | 0.81 (0.72-0.92) | <0.001 | 0.86 (0.75-0.98) | 0.022 | 0.87 (0.77-0.99) | 0.045 |
| **DI-GM_Unfavorable** | 1.00 (0.76-1.31) | 0.989 | 0.90 (0.68-1.21) | 0.498 | 0.94 (0.69-1.29) | 0.716 |

Model 1: Unadjusted (crude model)

Model 2: Adjusted for age, sex, race, education level, marital status, PIR

Model 3: Adjusted for age, sex, race, education level, marital status, PIR, drinking status, smoking status, CKM syndrome stages, BMI, white blood cell count, uric acid level

HRs and 95% CIs for all-cause and cardiovascular mortality per 1-point increase in DI-GM total, beneficial, and unfavorable scores, based on sensitivity analysis excluding deaths that occurred within the first two years of follow-up.
